# Continuous serratus posterior superior intercostal plane block for postoperative analgesia management in the patient who underwent right atrial mass excision: a case report

**DOI:** 10.1186/s12871-024-02535-4

**Published:** 2024-04-25

**Authors:** Ayşe Nurmen Akin, Yahya Yildiz, Selcuk Alver, Bahadir Ciftci

**Affiliations:** Department of Anesthesiology and Reanimation, Istanbul Medipol University, Mega Medipol University Hospital, No: 1 Bağcılar, Istanbul, 34040 Turkey

**Keywords:** Serratus posterior intercostal plane block, Postoperative analgesia management, Continuous infusion, Minimally invasive cardiac surgery

## Abstract

Serratus posterior intercostal plane block (SPSIPB) is a novel periparavertebral block. It provides anterolateral posterior chest wall analgesia. It is an interfascial plane block, performed under ultrasound guidance, and the visualization of landmarks is easy. It is performed deep into the serratus posterior superior muscle at the level of the third rib. Until now, there have been case reports about the usage of single-shot SPSIPB, but there are no reports about the usage of the block catheterization technique of SPSIPB. Continuous infusion from a catheter of interfascial plane blocks is important for postoperative analgesia management after painful surgeries such as thoracic and cardiac surgeries. Thus, we performed SPSIPB catheterization in a patient who underwent right atrial mass excision with minimally invasive thoracotomy surgery. Here, we present our successful analgesic experience with continuous SPSIPB in this case report.

## Background

Open heart surgery using median sternotomy is the gold standard treatment for patients with intracardiac masses [1]. However, a median sternotomy may cause complications, such as pain, wound infection, and prolonged hospitalization. Consequently, minimally invasive and percutaneous methods have been developed to reduce surgical invasiveness [1]. Minithoracotomy is a minimally invasive percutaneous procedure that significantly reduces the incidence of complications related to a standard median sternotomy. Bilateral minithoracotomies performed in minimally invasive cardiac surgery (MICS) can help significantly reduce the risk of wound infection [1]. However, despite being less invasive, patients undergoing a minithoracotomy experience severe postoperative pain [2].

For minimally invasive surgeries, the anesthetic approach should also involve the least invasiveness. For this purpose, regional anesthesia techniques are preferred over opioid use for postoperative pain management to prevent opioid-related side effects, such as nausea, vomiting, respiratory depression, constipation, and urinary retention. Serratus posterior superior intercostal block (SPSIPB) is a recent nerve-block technique described by Tulgar et al. in 2023 [3]. It involves applying an ultrasound (USG)-guided local anesthetic to the fascial plane between the serratus posterior superior and intercostal muscles to provide analgesia from the C3 to T10 levels. A case report by Bilal et al. described the efficacy of SPSIPB in patients undergoing MICS [4]. Notably, the deeply located serratus posterior superior muscle is also a potential site for catheterization; however, there is no existing evidence supporting the role of SPSIPB in catheterization. In this paper, we describe a case of successful postoperative pain management using continuous SPSIPB application in a patient undergoing MICS for right atrial mass excision.

## Case presentation

A 51-year-old male patient (weight = 128 kg; height = 180 cm) diagnosed with esophageal cancer was admitted to our hospital’s cardiovascular surgery outpatient clinic with a 3-week history of leg swelling; he did not have any other chronic disease. After appropriate investigations, a right atrial intracardiac mass was detected, and a MICS was planned for the patient. Before surgery, the SPSIPB procedure was explained in detail to the patient and his relatives, and verbal and written informed consent was obtained. After standard ASA monitoring (measurement of noninvasive blood pressure, 3-lead electrocardiogram, and oxygen saturation), general anesthesia was induced with midazolam (3 mg), propofol (2 mg/kg), fentanyl (2 mcg/kg), and rocuronium (0.6 mg/kg). Subsequently, the patient underwent orotracheal intubation using a size 8 cuffed endotracheal tube. The patient underwent minimally invasive right atrial mass excision and tricuspid valve repair in a minimally functioning heart, and a thoracic drain was inserted at the level of the right thoracic 6th intercostal space; the surgery lasted 3 h and 47 min. As per our hospital’s operating room multimodal analgesia protocol, we administered ibuprofen (400 mg) and tramadol (100 mg) intravenously (IV) 30 min before the end of the operation. No perioperative complications were observed.

**Fig. 1:**
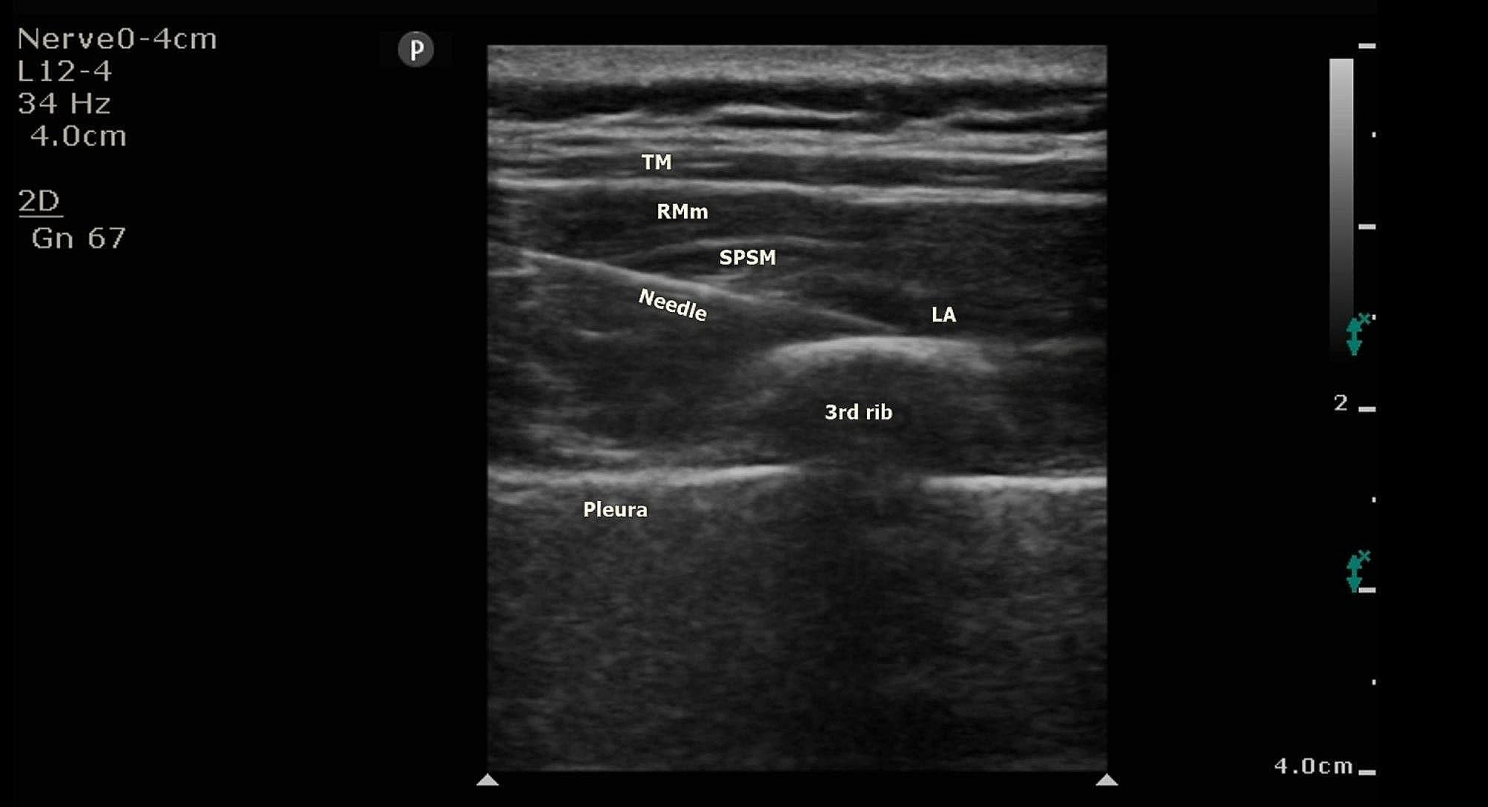
Sonographic anatomy of SPSIPB and spread of LA are seen. The spread of LA is between the rib and SPSM. The landmarks: TM, RMm, SPSM, 3rd rib, and pleurae are seen in the image. TM; Trapezius muscle, RMm; Rhomboid major muscle, SPSM; Serratus posterior superior muscle, LA; Local anesthetic

We did not extubate the patient after the surgery; he was transferred to the cardiovascular intensive care unit (CICU), where he was supposed to undergo SPSIPB catheterization while being intubated. For the catheterization, the patient was placed in the lateral decubitus position. Antisepsis was performed with chlorhexidine, and a linear USG probe was placed on the spine of the scapula using an in-plane technique and moved medially. The 3rd rib was identified just on the medial scapular border. After visualizing the trapezius, rhomboid major, and serratus posterior superior muscles (from top to bottom) under USG guidance, a 22-G X 80-mm block needle was directed caudally and advanced until it contacted the rib. To confirm and obtain a plane between the serratus posterior superior and intercostal muscles, hydrodissection was performed using 5 ml of isotonic solution. After the needle site was confirmed, 30 ml of 0.25% bupivacaine was injected (Fig. 1); the local anesthetic spread was confirmed by USG. Next, an 80 mm block catheter was inserted into this hydrodissected space to ensure analgesic continuity. The catheter tip was confirmed to lie between the serratus posterior superior and intercostal muscles using USG; the catheter was fixed to the skin with silk plaster. the patient remained cooperative through the entire CICU stay and was extubated one hour after SPSIPB administration. Patient-controlled analgesia (PCA) was attached via a block catheter (0.25% bupivacaine: 6 ml/h infusion, 4 ml/h bolus, 30 min lock-out interval). The infusion was maintained for 48 h.

After extubation, the patient’s static and dynamic (during coughing) pain scores (using a Numerical Rating Scale (NRS)), opioid and rescue analgesic needs, side effects, and complications were recorded every two hours. If the NRS score was > 4 at any hour, tramadol (1 mg/kg) was planned for rescue analgesia. Additionally, the Quality of Recovery-15 (QoR-15) questionnaire was administered to the patient at the 24th postoperative hour. IV paracetamol (1 g) was given every 8 h as a routine analgesic.

The highest NRS score reported by the patient was 3 during the entire 24 h postoperatively (Table 1). He was transferred to the ward after 24 h of ICU stay. The QoR-15 was performed in the CICU before transfer to the ward 24 h (Table 2). No additional rescue analgesia was administered to the patient during these 48 h. No side effects (itching, nausea, vomiting, or allergic reaction) or complications (pneumothorax, hematoma, or local anesthetic toxicity) were observed at the end of the 48-hour follow-up, so the block catheter was withdrawn.

**Table 1:** 24-hour NRS monitoring

Hour	Static NRS	Dynamic NRS
After extubation	0	0
2nd hour	0	3
4th hour	0	3
8th hour	0	3
10th hour	0	1
24th hour	0	0
36th hour	0	0
48th hour	0	1

**Table 2:** QoR-15T scoring of the patient at the postoperative 24 h

	Score
QoR-15T	141
Physical Comfort	48
Emotional State	38
Psychological support	18
Physical Independence	17
Pain	20

## Discussion and conclusions

This case report describes successful postoperative pain management using an SPSIPB block catheter in a patient undergoing minithoracotomy. Although the use of minithoracotomy has reduced the incidence of many complications associated with open surgeries requiring large incisions, there are certain drawbacks, such as increased intraoperative time and additional pain in the early postoperative period due to the involvement of the intercostal nerves and excessive rib retraction.

A minithoracotomy incision is made through the 2nd, 3rd, or 4th intercostal spaces with the patient in the supine position, and it can be unilateral or bilateral as per the surgical procedure [1]. With the widespread use of USG-guided regional anesthesia, there is a reduced need for opioid use and, consequently, a lower risk of opioid-related side effects. Perioperative pain management is a key factor in enhancing recovery after cardiac surgery in terms of minimizing mortality and morbidity, including the risks of downstream chronic pain [2].

Several regional anesthesia techniques are available for analgesia management after cardiothoracic surgery. Toscano et al. described the application of a USG-guided continuous deep serratus anterior plane block (SAPB) in patients undergoing minimally invasive mitral valve surgery via a right thoracotomy [5, 6]. SAPB blocks the lateral cutaneous branches of the intercostal nerves arising from T2–T9 by injecting the anesthetic into the fifth intercostal space in the mid-axillary line using the in-plane technique; this provides adequate analgesia for the anterolateral part of the chest. In contrast, SPSIPB provides analgesia in the anteroposterior region [3, 4], and thus was preferred in our study over SAPB.

Another technique for postoperative analgesia management after cardiothoracic surgery is the pectointercostal fascial plane block (PIFPB), which was published in 2014; it provides analgesia in the anterior part of the thorax and sternum [2]. Analgesia of the anterior cutaneous branch of the intercostal nerves is achieved by injecting a local anesthetic between the pectoralis major and the external intercostal muscles. Notably, the T6 level does not fully provide analgesia. A recent study has reported that local anesthesia applied between the serratus posterior and the intercostal muscles affects the lateral cutaneous nerve branches between the C3 and T7 levels, as well as the dorsal rami of the intercostal nerves [3]. It also provides analgesia in the anterolateral hemithorax in dermatomes corresponding to the C3–T10 levels. Therefore, we used SPSIPB instead of PIFPB in our patient.

Ciftci et al. performed SPSIPB in three patients undergoing breast surgery and reported dermatomes between C3 and T10 in dermatome analysis; they achieved adequate analgesia in these dermatomes [7]. Nevertheless, Ciftci et al. applied SPSIPB for VATS in a case series of 13 patients and achieved effective analgesia [8]. Plane block catheterization provides longer analgesia; however, there are no reports on SPSIPB catheterization in the literature. In light of this information in the literature, we applied SPSIPB to our patient, which also provided longer analgesia management without causing any complications.

Quality of recovery (QoR) after anesthesia and surgery are important concerns after any surgery [9]. The QoR after surgery depends on several factors. QoR testing objectively evaluates all aspects of postoperative recovery in the patient. For this purpose, we used the QoR-15T in our patient. In the first 48 h postoperatively, pain scores remained low, the QoR-15 T score was 141 points, and there was no need for opioids during this period. All these findings, which are especially relevant in the postoperative recovery of a patient undergoing cardiac surgery, highlight the efficacy of SPSIPB as an analgesic technique.

During SPSPB, caution must be exercised to not inject the local anesthetic into the spinal accessory nerve, which runs between the trapezius muscle and the rhomboid minor muscles; this may cause motor block of the trapezius muscle. Likewise, attention should be paid to the dorsal scapular artery and the dorsal scapular nerve that are in close proximity in this region.

The potential safety and efficacy of SPSIPB as a regional analgesia technique to achieve sensorial blockade between the C3 and T7/10 dermatomes and analgesia in the anterolateral posterior chest wall were the chief reasons for using SPSIPB in our patient. So far, there is no published literature regarding SPSIPB catheterization; hence, we aimed to assess the efficacy of this novel nerve block catheter technique.

In conclusion, SPSIPB is an effective regional anesthesia technique that can be used safely in MICSs. It provided effective analgesia and reduced the amount of opioids without causing any complications. Catheterization allows effective analgesia not only postoperatively but also during the later stages of postoperative follow-up, thereby accelerating the patient’s recovery and ensuring early discharge. Proper experimental trials are required since ours is just a case report.

## Data Availability

The datasets generated and/or analyzed during the current case report are not publicly available, but are available from the corresponding author on reasonable request.
